# Invasive Sphenoid Sinus Aspergillosis in a Patient With Chronic Neutropenia: A Case Report

**DOI:** 10.7759/cureus.92723

**Published:** 2025-09-19

**Authors:** Hafiz Fadl, Nicolas Bakinde, Teddy Ikhuoriah

**Affiliations:** 1 Internal Medicine, Grady Memorial Hospital, Atlanta, USA; 2 Internal Medicine, Morehouse School of Medicine, Atlanta, USA

**Keywords:** amphotericin b, case report, fungal sinusitis, immunocompetent host, sinus aspergillosis, sphenoid sinus, voriconazole

## Abstract

Invasive sinus aspergillosis is an uncommon but potentially life-threatening infection typically observed in immunocompromised individuals. However, increasing reports highlight its occurrence in patients with transient or borderline immunosuppression. We present a case of a 75-year-old female with treated mantle cell lymphoma and chronic neutropenia who developed invasive sphenoid sinus aspergillosis. Diagnosis was confirmed via imaging and histopathology following surgical intervention. She was treated with liposomal amphotericin B, followed by a successful transition to oral voriconazole, with full clinical recovery. This case underscores the importance of considering fungal sinusitis in patients without classical or sustained immunosuppression and supports a multidisciplinary, stepwise approach to management.

## Introduction

Invasive fungal sinusitis (IFS) is a rare but potentially lethal condition most commonly caused by *Aspergillus *spp. Although it traditionally affects severely immunocompromised patients, such as those with prolonged neutropenia or uncontrolled diabetes, it is increasingly recognized in individuals with partial or transient immune deficits, including those recovering from chemotherapy or receiving corticosteroids [1,2]. Clinical manifestations often mimic bacterial sinusitis, and delayed diagnosis may lead to serious complications, including bony erosion or intracranial invasion. This report presents an unusual case of isolated sphenoid sinus aspergillosis with bony erosion in a patient with chronic neutropenia and emphasizes the need for heightened clinical suspicion in borderline immunocompromised hosts [3-5].

## Case presentation

**Figure 1:**
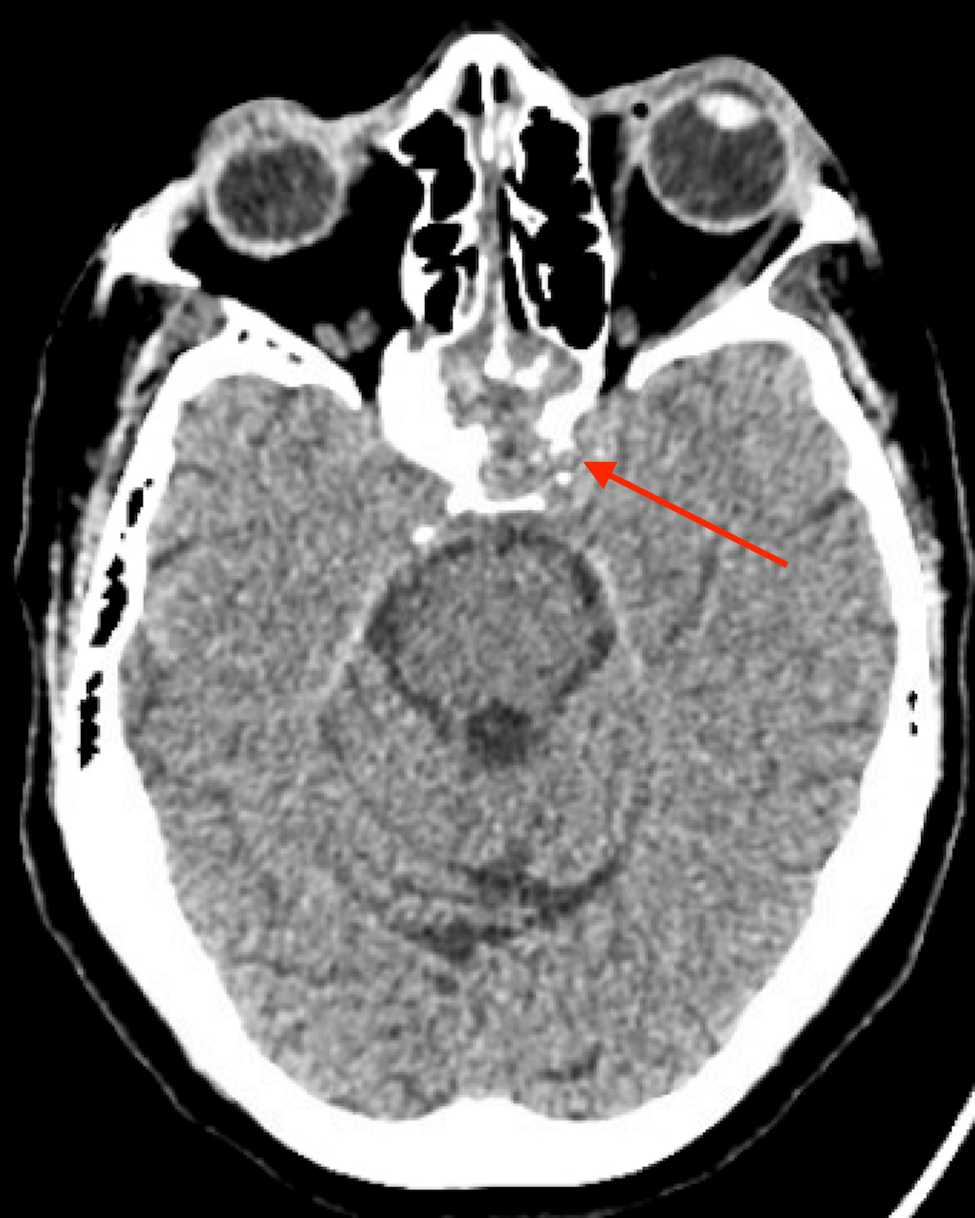
Axial CT image showing complete opacification of the bilateral sphenoid sinuses CT head without contrast demonstrates opacification of the sphenoid sinuses with associated osteogenesis and dehiscence of the posterior wall of the sphenoid sinus along the anterior wall of the sella.

A 75-year-old female with a history of hypertension, migraines, gastric cancer (status post-gastrectomy and chemotherapy in 2009), and mantle cell lymphoma (treated from September 2022 to January 2024) presented with worsening headaches over three months. She had recently completed a short course of corticosteroids for presumed inflammatory headaches. Her lymphoma treatment had been discontinued due to persistent neutropenia. On presentation, she was afebrile with stable vitals and a non-focal neurological exam. Head computed tomography (CT) without contrast showed opacification and bony erosion of the bilateral sphenoid sinuses without signs of orbital or intracranial involvement (Figure 1).

The Otolaryngology-Head and Neck Surgery team performed bilateral endoscopic sphenoidotomy. The procedure was conducted using stereotactic, computer-assisted cranial navigation under general anesthesia.

**Figure 2:**
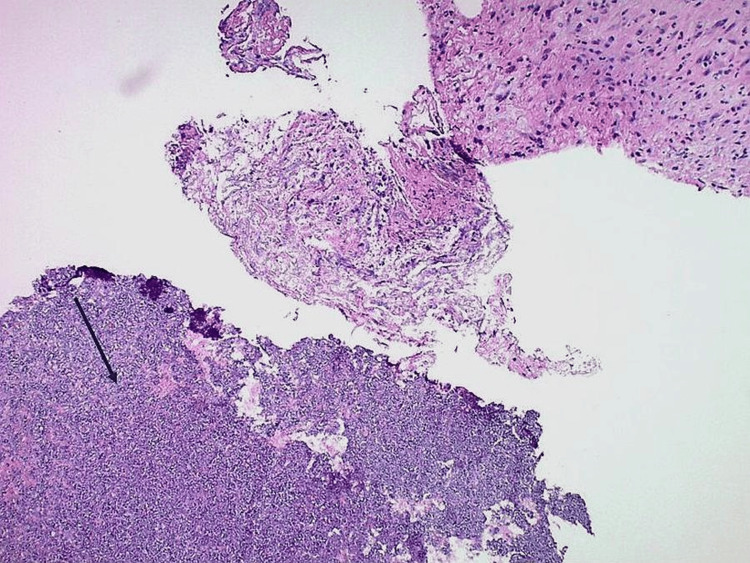
Hematoxylin and eosin–stained section of sphenoid tissue at 100× magnification Hematoxylin and eosin staining demonstrates necrotic tissue with a mixed inflammatory infiltrate and detached fungal elements (arrow).

**Figure 3:**
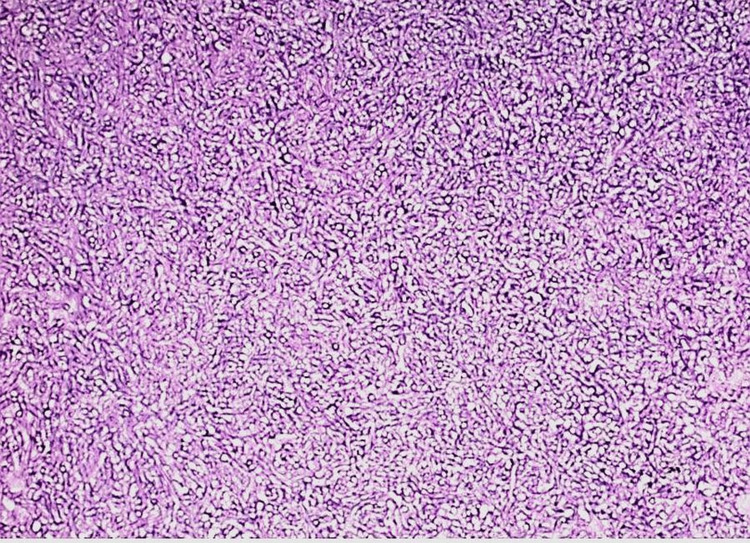
Hematoxylin and eosin-stained section of sphenoid tissue at 200× magnification Hematoxylin and eosin staining demonstrates numerous fungal hyphae with characteristic 45-degree branching, forming a compact fungal ball.

Intraoperative findings showed bilateral sphenoid sinuses filled with purulent drainage and thick fungal mucin, consistent with fungal sinusitis. Diffuse inflammatory changes were noted in the surrounding sinus mucosa. The left sphenoid sinus exhibited bony dehiscence at the skull base; however, there was no evidence of cerebrospinal fluid (CSF) leak. Histopathology revealed septate hyphae with acute-angle branching consistent with non-fumigatus *Aspergillus* species (Figures 2-3). Concurrent culture grew *Streptococcus pneumoniae*, indicating a polymicrobial infection.

The patient was initiated on intravenous liposomal amphotericin B at 3 mg/kg daily for 2 days, after which she was transitioned to oral voriconazole to complete a 12-week antifungal course. For the bacterial component, she received 2 days of intravenous ceftriaxone, followed by oral cefadroxil 500 mg every 12 hours for a total of 10 days. This regimen resulted in complete symptom resolution.

## Discussion

This case highlights the evolving epidemiology of invasive fungal sinusitis. Although our patient had completed chemotherapy several months earlier, her chronic neutropenia and recent corticosteroid exposure likely predisposed her to infection. The isolated sphenoid involvement with osseous erosion and polymicrobial infection is particularly rare and underscores the importance of early multidisciplinary evaluation and imaging, involving the Otolaryngology-Head and Neck Surgery, Radiology, Infectious Diseases, and Internal Medicine teams [6,7].

*Aspergillus fumigatus* is the most frequently isolated species in IFS. Diagnosis typically relies on radiographic features, such as sinus opacification and bony destruction, endoscopic visualization, and histopathological confirmation. Standard treatment involves prompt surgical debridement in combination with systemic antifungal therapy [1,8,9].

The differential diagnosis included bacterial sinusitis and mucormycosis. Bacterial sinusitis is a more common cause of acute sinus infection; however, the invasive features seen on imaging, together with culture results, supported a fungal etiology [10]. Mucormycosis, another invasive fungal sinus infection, can present with overlapping clinical and radiographic findings [11]. In this case, the absence of angioinvasive features, such as tissue necrosis, along with the identification of *Aspergillus* on microbiological analysis, distinguished it from mucormycosis [12,13]. These distinctions were critical in guiding the antifungal management strategy.

Voriconazole is the preferred agent due to its improved efficacy and safety profile compared with amphotericin B [1,8,14]. In this case, amphotericin was selected initially because of disease severity and diagnostic uncertainty, with a successful transition to voriconazole once the fungal etiology was confirmed. The polymicrobial nature of this infection, involving both *Aspergillus* and *Streptococcus pneumoniae*, did not alter the antifungal strategy, as *Aspergillus *was considered the primary pathogen. The bacterial component was managed with a short course of antibiotics, which resolved the secondary infection without influencing the antifungal regimen or overall clinical outcome. Table 1 shows a comparison of the present case with selected reports in the literature.

**Table 1:** Comparison of the present case with selected reports of invasive fungal sinusitis in immunocompetent or borderline immunocompetent patients

Author (Year)	Immune Status	Location Involved	Treatment	Outcome
Pushker et al. (2011) [15]	Immunocompetent	Orbit + paranasal sinuses	Surgery + amphotericin	Recovered
Vazquez et al. (2016) [6]	Borderline (ICU/critical illness)	Paranasal sinuses	Voriconazole monotherapy; combination therapy	Recovered
Apostolopoulou et al. (2020) [2]	COVID-associated	Pulmonary + paranasal sinuses	Voriconazole	Recovered
Current Case (2025)	Borderline (chronic neutropenia)	Isolated sphenoid sinus	Surgery + amphotericin B + voriconazole	Recovered

This case also highlights the value of a multidisciplinary approach involving Internal Medicine, Otolaryngology-Head and Neck Surgery, Infectious Diseases, and Pharmacy [16]. Timely evaluation by the Otolaryngology-Head and Neck Surgery team enabled prompt surgical debridement, while Pharmacy support ensured appropriate antifungal dosing and monitoring, both of which contributed to a favorable outcome.

## Conclusions

Sinus aspergillosis should be considered in elderly patients with chronic or treatment-resistant sinusitis, even when overt immunosuppression is not apparent. Patients with a history of chemotherapy, persistent neutropenia, or immunomodulatory treatment may be at elevated risk despite appearing immunocompetent. A high index of suspicion, timely imaging, surgical intervention, and appropriate antifungal therapy are essential for favorable outcomes. Increased clinician awareness of this underrecognized presentation may improve diagnostic and therapeutic timelines.
